# Compression of high-density EMG signals for trapezius and gastrocnemius muscles

**DOI:** 10.1186/1475-925X-13-25

**Published:** 2014-03-10

**Authors:** Cinthia Itiki, Sergio S Furuie, Roberto Merletti

**Affiliations:** 1 Biomedical Engineering Laboratory, Department of Telecommunications and Control Engineering, Escola Politecnica, University of Sao Paulo, Sao Paulo, Brazil; 2 Laboratorio di Ingegneria del Sistema Neuromuscolare, Department of Electronics, Politecnico di Torino, Torino, Italy

## Abstract

**Background:**

New technologies for data transmission and multi-electrode arrays increased the demand for compressing high-density electromyography (HD EMG) signals. This article aims the compression of HD EMG signals recorded by two-dimensional electrode matrices at different muscle-contraction forces. It also shows methodological aspects of compressing HD EMG signals for non-pinnate (upper trapezius) and pinnate (medial gastrocnemius) muscles, using image compression techniques.

**Methods:**

HD EMG signals were placed in image rows, according to two distinct electrode orders: parallel and perpendicular to the muscle longitudinal axis. For the lossless case, the images obtained from single-differential signals as well as their differences in time were compressed. For the lossy algorithm, the images associated to the recorded monopolar or single-differential signals were compressed for different compression levels.

**Results:**

Lossless compression provided up to 59.3% file-size reduction (FSR), with lower contraction forces associated to higher FSR. For lossy compression, a 90.8% reduction on the file size was attained, while keeping the signal-to-noise ratio (SNR) at 21.19 dB. For a similar FSR, higher contraction forces corresponded to higher SNR

**Conclusions:**

The computation of signal differences in time improves the performance of lossless compression while the selection of signals in the transversal order improves the lossy compression of HD EMG, for both pinnate and non-pinnate muscles.

## Background

In the medical field, compression techniques have been primarily applied to medical images, electrocardiography, and electroencephalography [1-7]. These techniques have also been applied to electromyography (EMG) signals [8-23].

Compression techniques applied to EMG signals belong to two main groups: transform-based and linear prediction methods. According to Guerrero and Mailhes [8], for EMG signal compression, discrete wavelet and cosine transforms would provide better results than linear prediction and pulse-code methods. In spite of this study, research on single-channel EMG-signal compression has been dealing with both linear prediction methods [9,10] and wavelet transforms [11-15]. Alternative approaches have also been applied to one-dimensional EMG signal [16-18], including the segmentation of a single EMG signal into matrix rows, followed by the use of two-dimensional techniques to the compression of a single-channel EMG signal [19-23].

The development of new wireless technologies for data transmission (e.g. Bluetooth), as well as the high-density multi-electrode arrays for EMG signal detection [24], has generated new research lines. In recent works, linear prediction techniques have been applied to the compression of multi-channel EMG signals from biceps brachii (BB) [25]. On the other hand, transform-based compression has not been applied to multi-channel EMG yet. Furthermore, no compression studies have been performed with multi-channel EMG from pinnate muscles, whose muscle fibers are not parallel to the skin surface and whose signals have different morphology.

In this work, compression techniques based on linear prediction (lossless JPEG) and transforms (lossy JPEG) were evaluated for multi-channel EMG signals from upper trapezius (UT) and medial gastrocnemius (MG). Each signal was placed on a matrix row, each sampling time instant was attributed to a column, and the matrix was interpreted as an image. Two signal arrangements provided different images to be compressed. The effect of applying compression before and after time differentiating the signals was also studied.

The aim of this research was to show the importance of methodological aspects on the use of lossless and lossy image compression techniques for propagating and non-propagating high-density multi-channel EMG signals.

## Methods

EMG signals differ from muscle to muscle and subject to subject, however the basic shapes and discharge rates of the constituent motor unit action potential (MUAP) trains are similar and 90-95% of the signal power is within the 10-450 Hz range. Muscle fiber conduction velocity (of the propagating components) is in the range 3-5 m/s and non-propagating components are generated by the “end of fiber effect” due to the extinction of the MUAPs at fiber ends. The latter phenomenon is particularly relevant in pinnate muscles whose fibers terminate near the surface.

The signals considered in this study were generated by three muscles: the Upper Trapezius (UT) and the Biceps Brachii (BB), both with fibers parallel to the skin and predominantly propagating components, and the Medial Gastrocnemius (MG) highly pinnate, with fiber at an angle with respect to the skin and predominantly non-propagating components.

### EMG signals from the upper trapezius muscle

**Figure 1:**
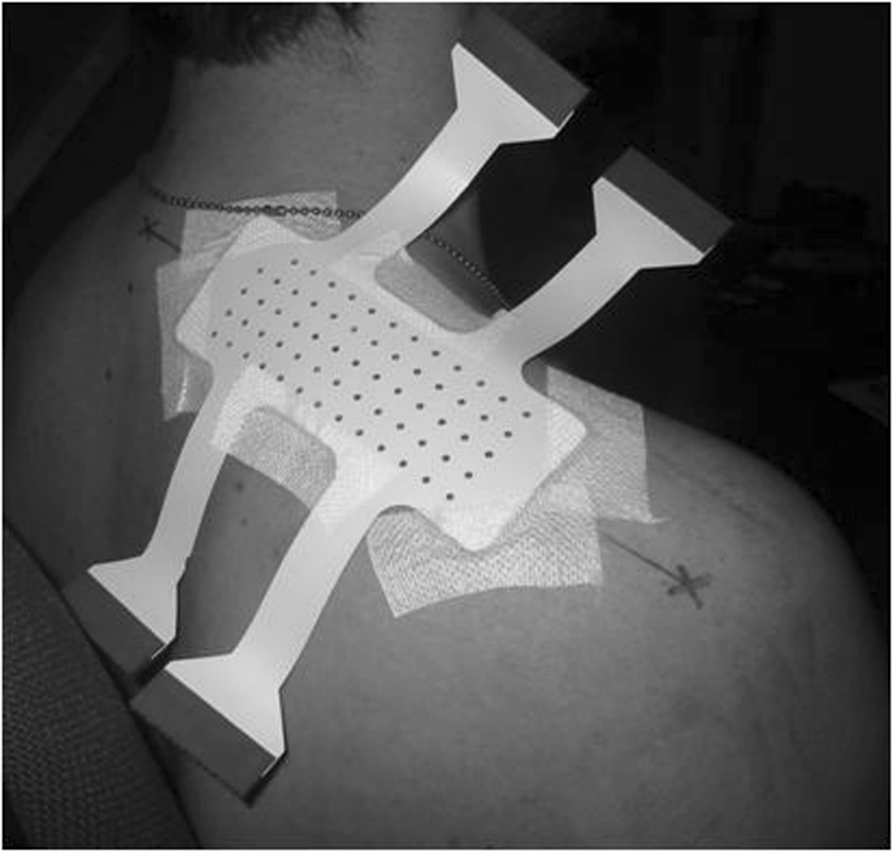
**Recording of upper trapezius single-differential signals.** Example of the 64 contact electrode matrix applied to the upper trapezius muscle.

Four recordings of 63 single-differential signals were obtained from a database from *Laboratorio di Ingegneria del Sistema Neuromuscolare* (LISiN), *Politecnico di Torino*, Italy [26]. All subjects signed an informed consent form and the protocol was approved by the local Regional Ethics Committee (Commissione di Vigilanza, Servizio Sanitario Nazionale – Regione Piemonte – ASL 1 – Torino, Italy). These signals were recorded from the UT muscle of two healthy males, at twenty and forty percent of maximum voluntary contraction force (MVC), using a two-dimensional surface-electrode matrix of sixty-four electrodes distributed in five rows and thirteen columns (no electrode in the left upper corner) [24], with rows positioned in the direction of muscle fibers (see Figure 1). Inter-electrode distance was 8 mm which satisfies the sampling theorem in space [27]. Single differential signals—given by the difference between two adjacent electrodes—were amplified and band-pass filtered (10Hz-750 Hz). Each signal *s*_*k*_(*t*) was acquired at a sampling frequency of 2,048 Hz per channel during 10s, and converted using a twelve-bit analog-to-digital converter. For processing, each 10s recording was divided into twenty 500 ms epochs.

### EMG signals from the medial gastrocnemius muscle

**Figure 2:**
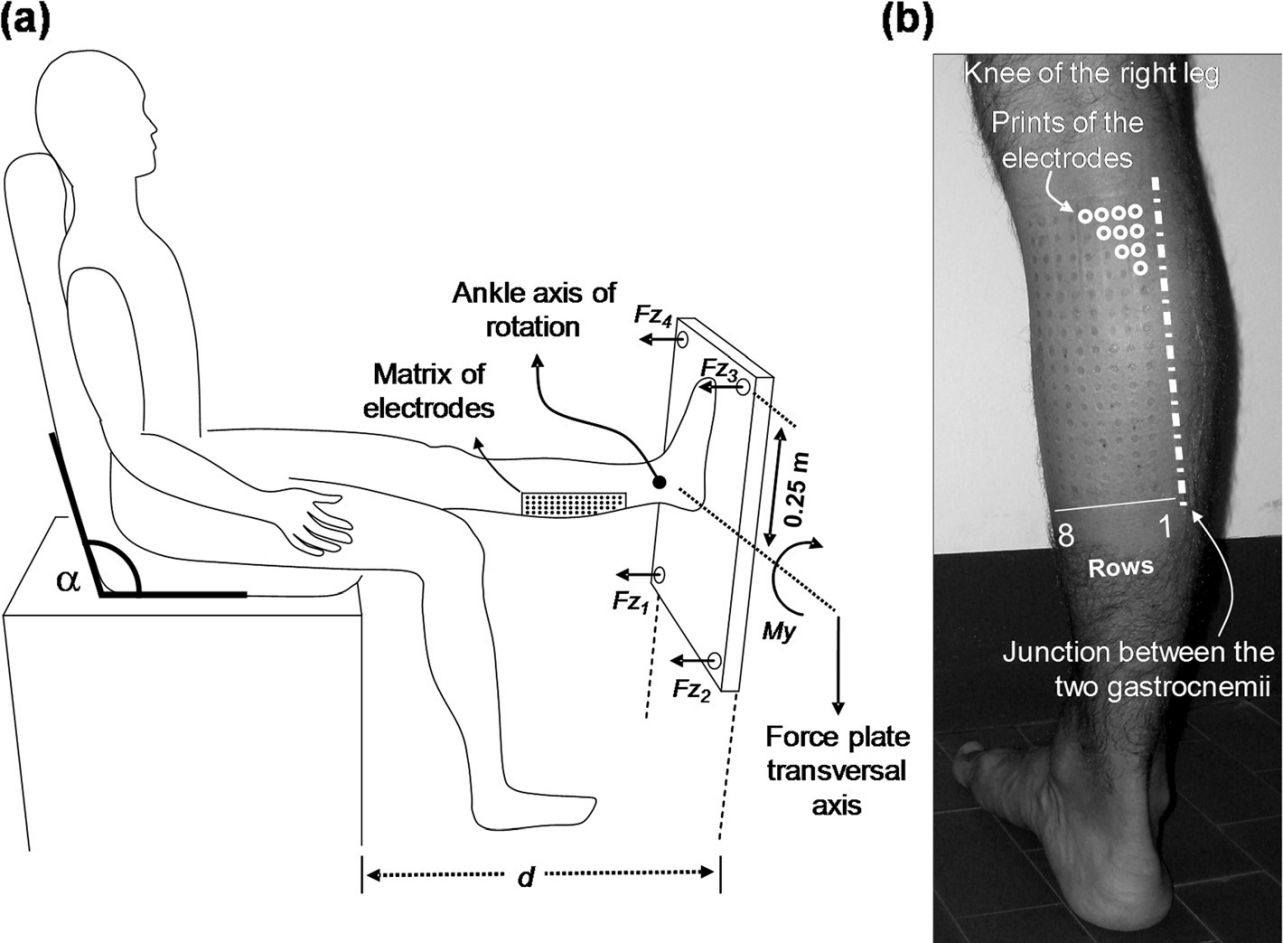
**Recording of medial gastrocnemius monopolar signals.** Recording of non-propagating monopolar EMG signals. **(a)** Schematic representation showing subject position. The ankle axis of rotation (i.e., the lateral malleolus) was aligned to the transversal axis of the force-plate. The plantar flexion torque (*My*) with respect to the force-plate transversal axis was computed as *My* = (*Fz*_*1*_ + *Fz*_*2*_ – *Fz*_*3*_ – *Fz*_*4*_)/4 and was displayed as a feedback to the subject. **(b)** The electrode matrix (8 rows and 16 columns) was positioned on skin regions covering exclusively the medial gastrocnemius muscle. The position was confirmed by ultrasound scanning. (courtesy of Dr. Taian Vieira, reproduced with permission [28]).

One recording of 128 monopolar signals was obtained from a database from *Laboratorio di Ingegneria del Sistema Neuromuscolare* (LISiN), *Politecnico di Torino*, Italy [28]. The subject provided written informed consent before volunteering to the fatiguing plantar-flexion protocol, which was approved by the local Ethics Committee. One healthy male subject was asked to isometrically increase his ankle plantar flexion torque in steps corresponding to twenty, forty, sixty and eighty percent of maximum voluntary contraction (MVC) force. Each contraction lasted approximately 5 s. These signals were recorded from the MG muscle with a two-dimensional surface-electrode matrix, with inter-electrode distance of 10 mm. One hundred and twenty eight electrodes were distributed in eight rows and sixteen columns, with rows parallel to the longitudinal axis of the muscle, as shown in Figure 2. Each monopolar signal corresponded to one electrode.

Differently from the case of the trapezius muscle, these signals are mostly non-propagating, because of the pinnate structure of the gastrocnemius muscle, and mostly due to the “end of fiber effect”.

Signals were amplified 500 times and band-pass filtered (10Hz-750 Hz). Each signal was acquired at a sampling frequency of 2,048 Hz per channel, using a twelve-bit analog-to-digital converter. Additional variables—such as forces and torques—were also recorded during the experiments, but did not belong to the scope of this study and therefore were not considered in this work. The recording was then divided into 500 ms epochs. Those epochs at the transition between different contraction force levels were discarded, so that each contraction level was, in the end, represented by eight epochs of 500 ms each. For lossless compression, 127 single-differential signals *s*_*k*_(*t*) were computed by the difference between adjacent monopolar signals.

### EMG signals from the biceps brachii muscle

Literature results [25] corresponded to EMG signals recorded from the BB muscle of seven subjects, at 50%MVC. A matrix of 61 electrodes—organized in thirteen rows and five columns without the four corner electrodes—was used. Inter-electrode distance was 5 mm. Signals were band-pass filtered from 10 Hz to 400 Hz, and acquired for 20 seconds at the rate of 1000 samples per second.

### Signal arrangements in images

Epochs were positioned in matrices, in which each row represented one of the MG or UT signals and each column corresponded to a time instant. Signal values were coded using grey levels, so each matrix could be interpreted as an image that corresponded to a 500 ms epoch of multi-channel EMG signals. The number of columns *M* was 1,024. The number of rows *N* was 63 for propagating single-differential signals (UT muscle), 127 for non-propagating single-differential signals (MG muscle), and 128 for non-propagating monopolar signals (MG muscle).

**Figure 3:**
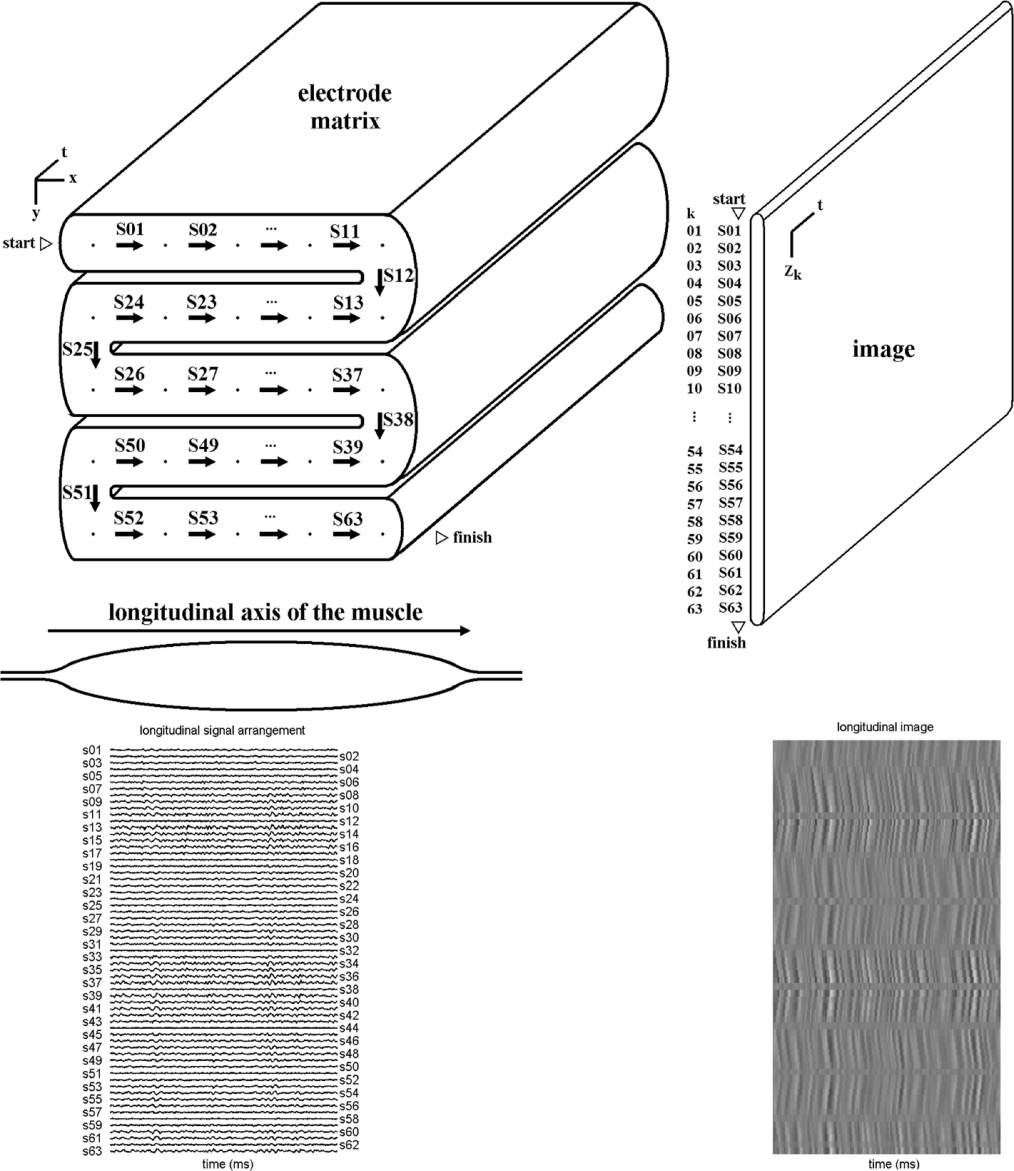
**Longitudinal arrangement of signals.** Longitudinal arrangement—signals are taken in the left-to-right (odd rows) and right-to-left (even rows) sequence from the electrode matrix. Rows are parallel to the longitudinal axis of the muscle. The beginning of the sequence and its end are indicated by triangles and the words “start” and “finish”. Arrows indicate how single-differential signals are taken as the differences between adjacent electrodes, which are represented by points. Each electrode corresponds to a monopolar signal. The image is formed by positioning one signal above the other, in the same sequence that they are taken from the electrode matrix.

**Figure 4:**
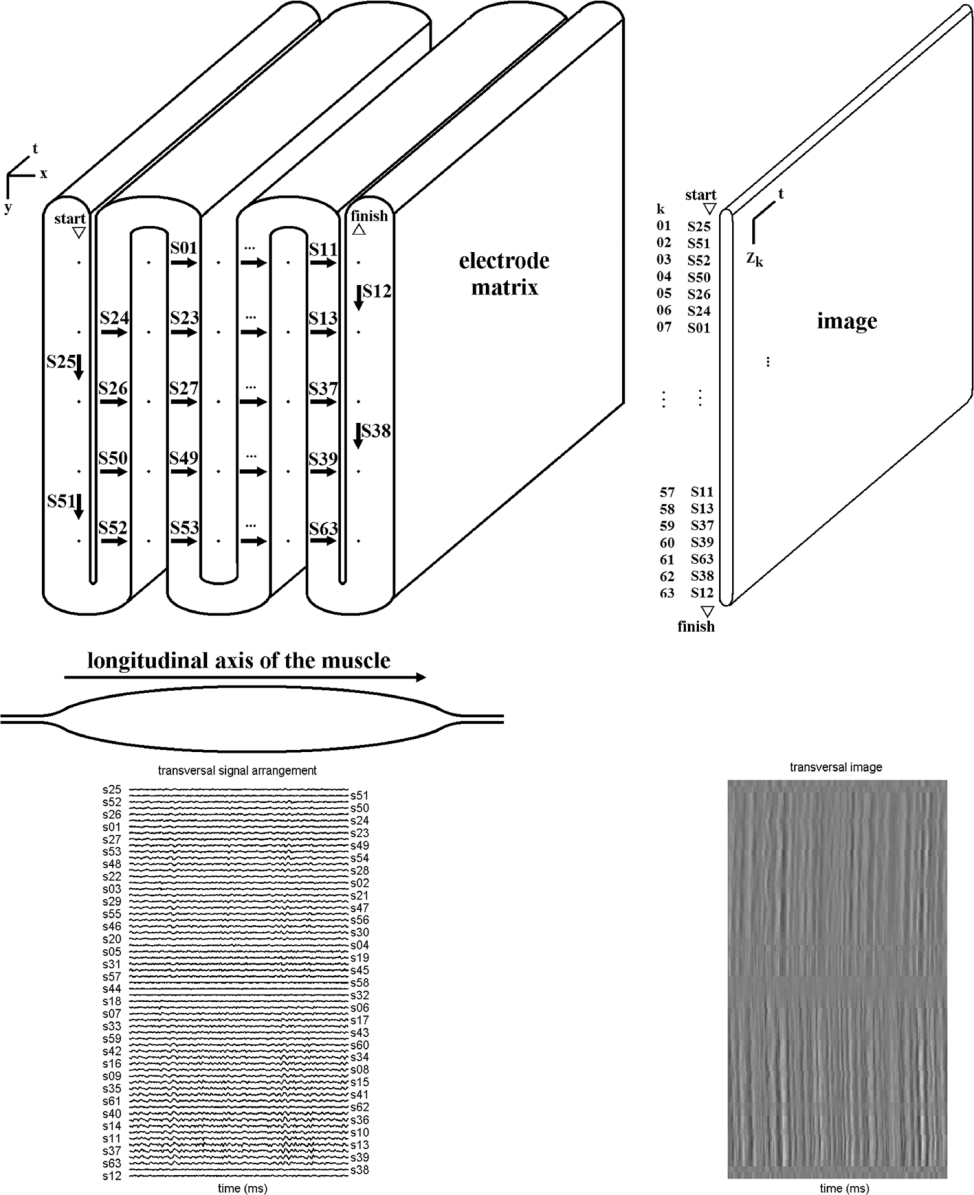
**Transversal arrangement of signals.** Transversal arrangement—signals are taken in the top-down (odd columns) and bottom-up (even columns) sequence from the electrode matrix. Rows are parallel to the longitudinal axis of the muscle. The beginning of the sequence and its end are indicated by triangles and the words “start” and “finish”. Arrows indicate how single-differential signals are taken as the differences between adjacent electrodes, which are represented by points. Each electrode corresponds to a monopolar signal. The image is formed by positioning one signal above the other, in the same sequence that they are taken from the electrode matrix.

The sequence in which signals were positioned in images followed two different orders—the “longitudinal arrangement” was parallel to the longitudinal axis of the muscle (see Figure 3), and the “transversal arragement” was perpendicular to it (as shown on Figure 4).

### Signal differentiation in time

Single-differential signals were differentiated with respect to time in order to take advantage from their time correlation, due to the limited signal bandwidth (10 Hz to 750 Hz). Then, compression was applied to the signal differences Δ_*k*_(*t*) = *z*_*k*_(*t*)-*z*_*k*_(*t*-*T*), where *T* was the sampling interval, *t* = *T*, …, 1024 *T* and *k* = 1, 2, …, *N*. In this way, each image row *k* represented one time-differentiated signal Δ_*k*_(*t*). In order to reconstruct the original signals without errors, the first image column included the original-signal samples Δ_*k*_(0) = *z*_*k*_(0), at time *t* = 0 ms.

**Figure 5:**
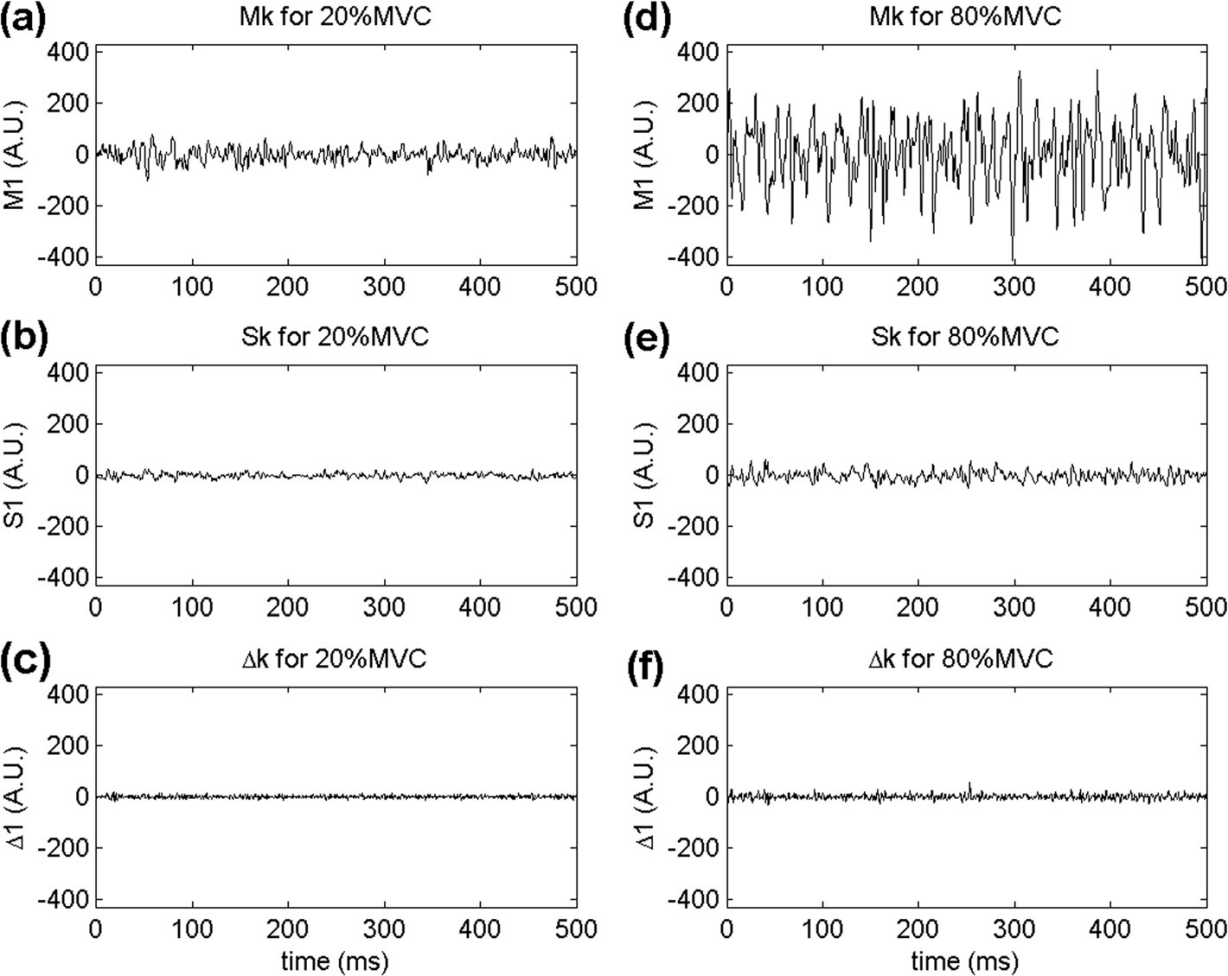
**Time differentiation and contraction forces.** Effect of time differentiation on two contraction forces: **a)** monopolar signal (*M*_*k*_), **b)** single-differential signal (*S*_*k*_) and **c)** time-differences (Δ_*k*_) of single-differential signal for 20%MVC; **d)***M*_*k*_, **e)***S*_*k*_ and **f)** Δ_*k*_ signals for 80%MVC.

Figure 5 shows single-differential *S*_*k*_ signals and their time-differences Δ_*k*_, for 20%MVC and 80%MVC. Monopolar signals *M*_*k*_ are also illustrated in Figure 5.

For compression purposes, multi-channel high-density EMG samples were represented by grey images. Two approaches were considered: lossless and lossy compression.

### Lossless compression

#### *Lossless JPEG*

A lossless image compression standard—established by the Joint Photographic Experts Group (JPEG)—was used in this work [29]. Since the source image could be reconstructed exactly from the decoding of the compressed image data, the original EMG samples could also be perfectly recovered.

In the lossless JPEG encoding, the value of the sample at position (*r*, *c*) is estimated by a predictor $\hat{y}\left(r,c\right)$ based on the values of three neighbor samples *y*(*r*–1,*c*), *y*(*r*,*c*–1) and *y*(*r*–1,*c*–1), where *r* is the number of the current row and *c* is the number of the current column. This is a causal template, a subset of the available past data. The difference between the actual value of the sample *y*(*r*,*c*) and the estimate $\hat{y}\left(r,c\right)$ is entropy coded by either Huffman or arithmetic coding, in a lossless fashion. This guarantees that the source image can be perfectly reconstructed from the decoding of the compressed image data. The decoding process occurs in an order that is reverse to the coding process [29].

#### *Lossless ZIP compression*

For comparison purposes, an alternative lossless method was used—the ZIP compression of files. ZIP is a public-domain standard that is widely used for file compression. It uses the DEFLATE algorithm [30] for lossless compression which, in turn, combines the Lempel and Ziv LZ77 algorithm [31] with Huffman coding [29].

In this work, samples from the A/D converter were initially represented by 12 bits. Since each byte has only 8 bits, a routine to convert two 12-bit samples into three 8-bit bytes was needed. The 212 format is a standard used in some PhysioBank data files [32] that represents two successive 12-bit samples— *b*_12_*b*_11_*b*_10_*b*_09_*b*_08_*b*_07_*b*_06_*b*_05_*b*_04_*b*_03_*b*_02_*b*_01_ and *c*_12_*c*_11_*c*_10_*c*_09_*c*_08_*c*_07_*c*_06_*c*_05_*c*_04_*c*_03_*c*_02_*c*_01_ —into a sequence of three 8-bit bytes— *b*_08_*b*_07_*b*_06_*b*_05_*b*_04_*b*_03_*b*_02_*b*_01_, *c*_12_*c*_11_*c*_10_*c*_09_*b*_12_*b*_11_*b*_10_*b*_09_, and *c*_08_*c*_07_*c*_06_*c*_05_*c*_04_*c*_03_*c*_02_*c*_01_ —where *b*_*i*_ and *c*_*i*_ are the *i*^th^ bit of the respective 12-bit samples.

The computed bytes were then saved in Matlab® uncompressed format (version 6), as unsigned 8-bit integers. These .mat files were later compressed in ZIP standard to provide a reference value for comparison purposes.

### Lossy compression

Lossy methods were applied to both longitudinal and transversal arrangements of the recorded signals.

#### *Lossy JPEG*

In lossy JPEG, the source image data are divided into blocks of 8-pixels by 8-pixels. Then the two-dimensional discrete cosine transform (DCT) is applied to each block, resulting in sixty four DCT coefficients. Each one of the DCT coefficients goes through a process known as quantization, in which it is divided by a value retrieved from a quantization table and then rounded off to the nearest integer. This quantization process inserts losses.

In the JPEG still picture compression standard specifications [29], methods implementing the standard should provide a wide range of image quality ratings—“moderate”, “good”, “very good”, “excellent” and “usually indistinguishable from the original”. The quality parameter (*q*) provided by Matlab’s imwrite funtion is a number between 0 and 100, that allows the user to control the compressed-image characteristics by associating higher values with higher quality, smaller image degradation, and larger file sizes.

In lossy JPEG, the quantization-table values are modulated by a number that is directly related to the value of the quality parameter. Quantization-table values are lower for higher values of the quality parameter. DCT coefficients are divided by smaller quantization-table values, with the consequent reduction in quantization errors and “image degradation”.

After quantization, coefficients are rearranged into a vector form, according to the order known as zig-zag order. For further compression, the resulting vector of DCT coefficients is entropy encoded by Huffman coding, according to a criterion provided in specific tables, referred to as “Table Specification” in [29].

#### *Linear prediction*

In previous research, Carotti and colleagues proposed a lossy compression technique based on linear prediction applied to single-channel EMG [10] and multi-channel EMG [25] signals (sixty one signals from a 5 × 13 matrix without the four corner electrodes).

In the case of modified Algebraic Code Excited Linear Prediction (ACELP), each EMG signal recorded from BB muscle was represented by parameters derived from an autoregressive model and a prediction residual signal. The residual signal was coded by a method that minimized the mean-squared reconstruction error. The quantization process was applied to the parameters and to the residual signal, so that a lossy compression was obtained [10,30-35]. ACELP has been applied to independent coding of single-channel EMG signals or to the spectral components. It has also been used in spatial and temporal prediction [25].

### Compression evaluation

Two compression aspects were evaluated in this work—data size and errors.

#### *Compression performance*

For UT muscle, the number of bytes required to store 500 ms of uncompressed single-differential signals was 96,768 bytes (63 channels × 1,024 samples/channel × 12 bits/sample/8 bits/byte). In the case of MG muscle, the uncompressed file size for each 500 ms epoch was 195,072 bytes for 127 single-differential signals, and 196,608 bytes for 128 monopolar signals.

Compression ratio (CR) is defined as the ratio between the uncompressed data size and the compressed data size:

$$\mathrm{CR}=\frac{\text{uncompressed}\;\text{file}\;\text{size}}{\text{compressed}\;\text{file}\;\text{size}}.$$

File size reduction (FSR) considers the size difference between original and compressed data, with respect to the original data size. It is usually given in percentage:

$$\mathrm{FSR}=100\%\times \frac{\text{uncompressed}\;\text{file}\;\text{size}-\text{compressed}\;\text{file}\;\text{size}}{\text{uncompressed}\;\text{file}\;\text{size}}=100\%\times \left(1-1/\mathrm{CR}\right)$$

For a specific experimental condition—muscle, contraction force level, arrangement type and compression method—compression performance parameters were computed for several individual epochs. In the case of UT muscle, each contraction force corresponded to forty 500 ms epochs, obtained from two 10s recordings. For MG muscle, eight 500 ms epochs were associated to each contraction force. For clarity reasons, instead of presenting results for all the individual epochs in the same experimental condition, we computed mean values and standard deviations of CR and FSR, and presented them in tables, alongside compression error measurements described below. However, to facilitate reading, only the mean values are mentioned in the text.

#### Compression errors

For lossy compression, the inverse process—decompression—results in reconstructed signals ${\hat{s}}_{k}\left(t\right)$ that are not exactly equal to the EMG signals *s*_*k*_(*t*). This process introduces errors that are defined as

$$e_{k}\left(t\right)=s_{k}\left(t\right)-{\hat{s}}_{k}\left(t\right).$$

The maximum absolute error (MAE) is the maximum value of the absolute error over all samples in the matrix:

$$\mathrm{MAE}=\max_{k,m}\left\{\left|s_{k}\left(\mathrm{mT}\right)-{\hat{s}}_{k}\left(\mathrm{mT}\right)\right|\right\},$$

where *k* = 1,…*N* is the signal number, *m* = 1, …1024 is the sample number, and *T* is the sampling time (inverse of the sampling frequency). In this work, MAE is given in arbitrary units (A.U.). The number of A.U. could correspond to the number of quantization levels, where the quantization level ΔV of an A/D converter is given by the input voltage range divided by the total number of quantization levels. It can also be associated to the non-amplified EMG, by dividing ΔV by the amplifier’s gain. For example, signals from both the UT and the MG muscles were converted using a twelve-bit A/D converter, whose input varied from -10 V to +10 V. Hence, the quantization level was 20 divided by 4,096 (2^12^), i.e., ΔV = 4.88 mV. One A.U. could be made equivalent to one ΔV. If the amplifier’s gain (500) is taken into account, one A.U. could also be associated to a value of (ΔV/gain) = 9.77 μV for the non-amplified EMG signal. However, the use of integers for A.U. is better suited for compression purposes than real voltage values. Therefore in this work, MAE may vary from 0A.U. to 4,096A.U.

The signal-to-error ratio (SNR) is given in decibels (dB) by:

$$\mathrm{SNR}=10\log_{10}\frac{\sum_{k=1}^{N}\sum_{m=1}^{M}s_{k}^{2}\left(\mathrm{mT}\right)}{\sum_{k=1}^{N}\sum_{m=1}^{M}{\left(s_{k}\left(\mathrm{mT}\right)-{\hat{s}}_{k}^{2}\left(\mathrm{mT}\right)\right)}^{2}},$$

where *N* = 63 (for propagating EMG signals) or *N* = 128 (for non-propagating monopolar signals) is the number of signals, and *M* = 1024 is the number of samples per channel corresponding to a 500 ms epoch.

Note that for lossless compression, maximum absolute error (MAE) is zero, while signal-to-noise error (SNR) is infinite, because the original signals can always be reconstructed without errors.

## Results

**Figure 6:**
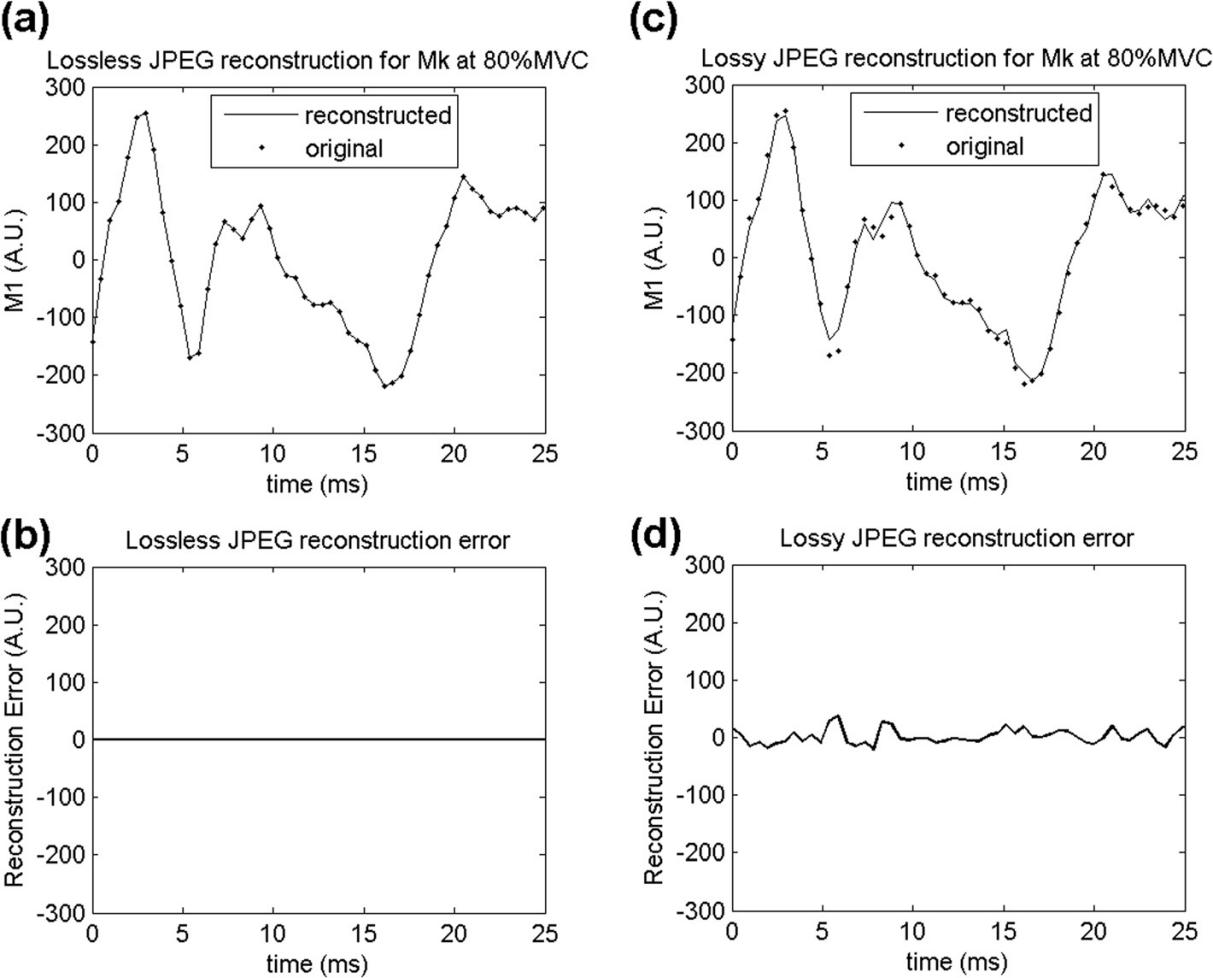
**Lossless and lossy reconstruction.** Short segment of original (dashed line) and reconstructed signals (continuous line) for **a)** lossless JPEG and **c)** lossy JPEG. The reconstruction error for **b)** lossless JPEG is always zero, while for **d)** lossy JPEG, it depends on the chosen value for the quality parameter. (*q* = 13 in this figure). For the first 500 ms monopolar signal (shown on the figure), MAE was 45 A.U. and SNR was 19.22 dB, while for the whole set of 128 monopolar signals, MAE was 101 A.U. and mean SNR was 19.88 dB. The compression performance was given by a FSR of 90.64% for lossy JPEG and 33.52% for lossless JPEG.

For illustration purposes, a short segment of the original and reconstructed signals *S*_1_ are shown on Figure 6, as well as reconstruction errors. For the longitudinal arrangement of the seventh 500 ms epoch at 80%MVC of MG muscle, the quality parameter (*q*) for lossy compression was chosen as 13, since it provides over 90% compression, while keeping a SNR around 20 dB. Considering all the 128 signals of the 500 ms epoch, the lossy compression performance was given by a FSR of 90.6%, a MAE of 101 A.U. and a SNR of 19.88 dB. Using lossless JPEG compression, FSR was 33.52% for this specific epoch.

Table 1 presents FSR results for lossless compression techniques—ZIP and lossless JPEG—for both pinnate (MG) and non-pinnate (UT) muscles.

**Table 1:** Lossless compression results

**Method**	**Signal**	**Arrangement**	**Muscle**	**% of MVC**			
				**20%**	**40%**	**60%**	**80%**
ZIP	*S*_*k*_	L	UT	17.98 ± 0.59	14.69 ± 0.66		
ZIP	*S*_*k*_	T	UT	19.78 ± 0.51	16.69 ± 0.67		
JPEG	*S*_*k*_	L	UT	36.72 ± 0.77	31.79 ± 0.79		
JPEG	*S*_*k*_	T	UT	36.74 ± 0.77	31.81 ± 0.78		
JPEG	Δ_*k*_	L	UT	40.86 ± 0.31	38.13 ± 0.40		
JPEG	Δ_*k*_	T	UT	40.88 ± 0.31	38.15 ± 0.41		
ZIP	*S*_*k*_	L	MG	37.32 ± 0.89	31.73 ± 0.56	27.32 ± 0.63	25.31 ± 0.66
ZIP	*S*_*k*_	T	MG	38.11 ± 0.82	32.94 ± 0.50	28.71 ± 0.59	26.78 ± 0.65
JPEG	*S*_*k*_	L	MG	59.29 ± 1.21	52.52 ± 0.68	47.77 ± 0.59	45.40 ± 0.76
JPEG	*S*_*k*_	T	MG	59.30 ± 1.20	52.53 ± 0.69	47.79 ± 0.59	45.41 ± 0.77
JPEG	Δ_*k*_	L	MG	58.31 ± 0.72	53.40 ± 0.53	49.88 ± 0.55	48.21 ± 0.67
JPEG	Δ_*k*_	T	MG	58.31 ± 0.72	53.41 ± 0.53	49.90 ± 0.55	48.22 ± 0.67

Lossless compression of single-differential (*S*_*k*_) signals and their differences in time (Δ_*k*_). Mean values ± standard deviation of file-size reduction (%) were computed for 40 epochs per contraction level (% of MVC) of upper trapezius (UT) signals and for 8 epochs per contraction level (% of MVC) of medial gastrocnemius (MG) signals. Arrangements were longitudinal (L) and transversal (T). Methods were ZIP and lossless JPEG.

### Lossless ZIP compression results

**Figure 7:**
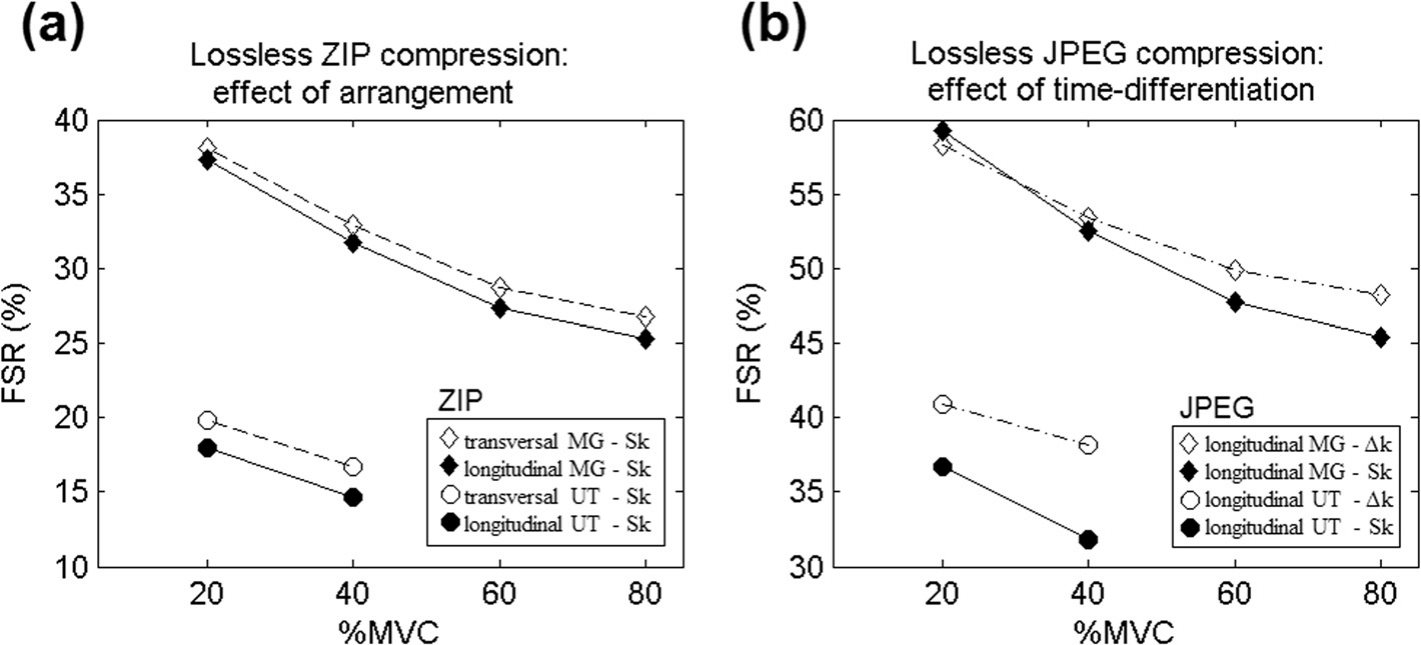
**Lossless compression.** File-size reductions (FSR) as functions of contraction level (%MVC). **a)** Lossless ZIP compression results are presented for the longitudinal (filled circles) and transversal (empty circles) arrangements of upper trapezius signals, and for the longitudinal (filled diamonds) and transversal (empty diamonds) arrangements of medial gastrocnemius signals. **b)** Lossless JPEG compression results are presented for single-differential upper trapezius signals before (filled circles) and after (empty circles) time differentiation, and for single-differential medial gastrocnemius signals before (filled diamonds) and after (empty diamonds) time differentiation.

ZIP compression provided the worst results for the compression of propagating and non-propagating single-differential signals. Figure 7a shows the ZIP compression results for the longitudinal and transversal arrangement of both non-pinnate and pinnate muscle signals.

#### Single-differential signals from UT muscle

For the longitudinal arrangement of propagating single-differential signals (UT muscle), average FSRs of 17.98% (for 20%MVC) and 14.69% (for 40%MVC) were obtained. Compared to the longitudinal arrangement, the transversal arrangement provided additional FSRs of 1.80% (for 20%MVC) and 2.00% (for 40%MVC) on average.

#### Single-differential signals from MG muscle

ZIP compression of non-propagating single-differential signals (MG muscle) provided average FSRs of 37.32% (for 20%MVC), 31.73% (for 40%MVC), 27.32% (for 60%MVC) and 25.31% (for 80%MVC). Compared to the longitudinal arrangement, the transversal arrangement provided additional FSRs of 0.79% (for 20%MVC), 1.21% (for 40%MVC), 1.39% (for 60%MVC), and 1.47% (for 80%MVC) on average.

### Lossless JPEG compression results

Lossless JPEG was applied to images of single-differential signals and their time-differences. Figure 7b shows FSR for the longitudinal arrangement. For FSR values in the range below 55.0%, there is an improvement in FSR if time differentiation is applied to single-differential signals before the lossless-JPEG compression.

#### Single-differential signals from UT muscle and time differences

Lossless JPEG was applied directly to images of propagating single-differential signals in the longitudinal arrangement. It resulted in average FSRs of 36.72% (for 20%MVC) and 31.79% (for 40%MVC). These values will be used as reference for the comparisons that follow. The transversal arrangement of single-differentiated signals did not bring any noticeable improvement on the FSR, for both contraction force levels. The process of performing time differentiation of single-differential signals, before applying the lossless JPEG compression, provided additional FSRs of 4.14% (for 20%MVC) and 6.34% (for 40%MVC) with respect to the mean values obtained for compressed single-differential signals, in both longitudinal and transversal arrangements.

#### *Single-differential signals from MG muscle and time differences*

Lossless JPEG was applied to the longitudinal arrangement of non-propagating single-differential signals and resulted in average FSRs of 59.29% (for 20%MVC), 52.52% (for 40%MVC), 47.77% (for 60%MVC) and 45.40% (for 80%MVC). The transversal arrangement did not bring any significant improvement on the FSR. For the longitudinal arrangement, time differentiation caused a decrease on average FSR of 0.98% (for 20%MVC). It also caused increases of 0.88% (for 40%MVC), 2.11% (for 60%MVC), and 2.81% (for 80%MVC) on average. Similar results were obtained for the transversal arrangement.

### Lossy JPEG compression results

**Figure 8:**
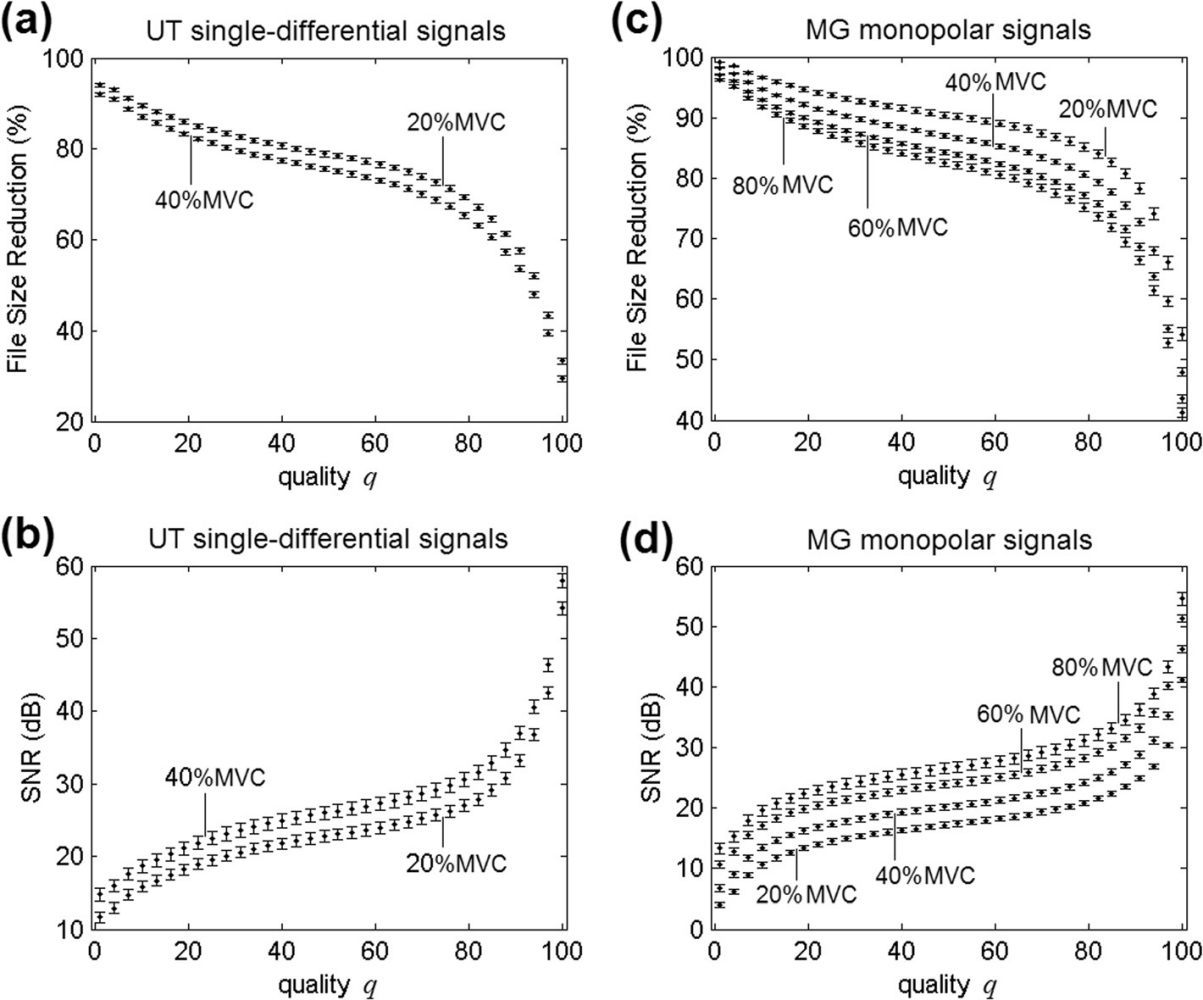
**Lossy compression.** File-size reduction (%) as a function of lossy JPEG quality parameter, for **a)** UT single-differential (*S*_*k*_) signals and **c)** MG monopolar (*M*_*k*_) signals. Signal-to-noise ratio (SNR) in decibels (dB) as functions of lossless JPEG quality parameter, for **b)** UT muscle and **d)** MG muscle. Mean values are represented by small filled circles and (mean values ± standard deviations) are shown by bars.

The lossy JPEG quality (*q*) parameter was varied within the 1 to 100 range. As a consequence, for each 500 ms epoch, one hundred compressed files were generated. Each file corresponded to one quality value, contraction force and muscle. The mean values of file size and signal-to-noise ratio were computed for similar epochs (same quality, contraction and muscle). Figure 8 shows how FSR and SNR vary with the quality parameter, for different contractions and muscles. In this figure, the transversal arrangement was used, since it provided the best results for both pinnate MG and non-pinnate UT muscles.

Table 2 shows the results for lossy JPEG compression—the FSR (%), the signal-to-noise ratio (dB) and the maximum absolute error (A.U.)—as well as the value of the quality parameter (*q*) used to obtain the corresponding mean FSR value. Signals were positioned in images, according to the longitudinal (L) or transversal (T) orders with respect to the muscle fiber direction. Signal types are non-propagating monopolar EMG signals from the MG muscle (N = 8 epochs), propagating single-differential EMG signals from UT muscle (N = 40 epochs, i.e., twenty epochs from 2 subjects) and propagating single-differential EMG signals from BB muscle.

**Table 2:** Lossy compression results

**Method**	**Mus.**	**Arr.**	**Var.**	**MVC**							
				**10%**	**20%**	**30%**	**40%**	**50%**	**60%**	**70%**	**80%**
ACELP	BB	L	FSR (%)	87.3		87.3			87.3	87.3	
[10]			SNR (dB)	10.38		11.81			12.25	12.79	
JPEG	UT	L	FSR (%)		87.5 ± 0.5		87.1 ± 0.4				
			SNR (dB)		17.17 ± 0.74		18.63 ± 0.86				
			MAE (A.U.)		134.7 ± 10.4		168.0 ± 13.8				
			q		15		10				
JPEG	UT	T	FSR (%)		87.3 ± 0.4		87.2 ± 0.4				
			SNR (dB)		19.15 ± 0.78		20.45 ± 0.80				
			MAE (A.U.)		109.3 ± 10.5		147.3 ± 22.8				
			q		19		13				
JPEG	MG	L	FSR (%)		87.3 ± 0.6		87.3 ± 0.4		87.3 ± 0.3		87.3 ± 0.4
			SNR (dB)		19.48 ± 0.42		19.98 ± 0.49		21.88 ± 0.59		23.33 ± 0.84
			MAE (A.U.)		25.4 ± 1.7		43.9 ± 5.7		63.0 ± 6.6		78.5 ± 5.4
			q		71		47		31		24
JPEG	MG	T	FSR (%)		87.3 ± 0.5		87.3 ± 0.3		87.3 ± 0.3		87.2 ± 0.5
			SNR (dB)		19.92 ± 0.37		20.99 ± 0.50		22.95 ± 0.58		24.24 ± 0.85
			MAE (A.U.)		21.4 ± 2.2		35.9 ± 2.0		51.8 ± 4.6		62.0 ± 5.3
			q		73		52		34		26
IC [25]	BB	L	FSR (%)					89.6			
			SNR (dB)					15.58			
JPEG	UT	L	FSR (%)		89.6 ± 0.4		89.5 ± 0.4				
			SNR (dB)		15.81 ± 0.72		17.17 ± 0.87				
			MAE (A.U.)		160.5 ± 13.6		189.5 ± 17.8				
			q		10		06				
JPEG	UT	T	FSR (%)		89.5 ± 0.4		89.6 ± 0.4				
			SNR (dB)		17.66 ± 0.75		18.87 ± 0.82				
			MAE (A.U.)		129.4 ± 12.7		170.0 ± 26.1				
			q		13		08				
JPEG	MG	L	FSR (%)		89.6 ± 0.5		89.5 ± 0.3		89.7 ± 0.3		89.5 ± 0.4
			SNR (dB)		17.84 ± 0.42		18.39 ± 0.49		20.09 ± 0.58		21.63 ± 0.83
			MAE (A.U.)		30.8 ± 2.3		60.0 ± 7.2		78.3 ± 8.5		94.6 ± 8.3
			q		57		32		20		16
JPEG	MG	T	FSR (%)		89.6 ± 0.4		89.6 ± 0.3		89.7 ± 0.3		89.5 ± 0.4
			SNR (dB)		18.37 ± 0.37		19.39 ± 0.50		21.14 ± 0.55		22.36 ± 0.83
			MAE (A.U.)		26.4 ± 2.0		39.6 ± 1.6		67.5 ± 5.7		75.4 ± 6.8
			q		60		35		22		17
SP [25]	BB		FSR (%)					91.0			
			SNR (dB)					15.55			
STP[25]	BB		FSR (%)					91.0			
			SNR (dB)					18.96			
JPEG	UT	L	FSR (%)		91.1 ± 0.4		90.9 ± 0.4				
			SNR (dB)		14.72 ± 0.71		15.94 ± 0.87				
			MAE (A.U.)		176.7 ± 13.8		212.2 ± 12.9				
			q		7		4				
JPEG	UT	T	FSR (%)		91.2 ± 0.4		90.8 ± 0.4				
			SNR (dB)		16.44 ± 0.75		17.94 ± 0.83				
			MAE (A.U.)		148.6 ± 12.6		179.5 ± 19.0				
			q		9		6				
JPEG	MG	L	FSR (%)		91.0 ± 0.5		91.0 ± 0.3		91.1 ± 0.3		90.9 ± 0.4
			SNR (dB)		16.74 ± 0.42		17.23 ± 0.48		18.92 ± 0.59		20.39 ± 0.83
			MAE (A.U.)		38.4 ± 3.9		61.0 ± 4.1		91.0 ± 9.8		113.5 ± 10.3
			q		44		24		15		12
JPEG	MG	T	FSR (%)		91.0 ± 0.4		90.9 ± 0.3		90.9 ± 0.3		90.8 ± 0.4
			SNR (dB)		17.34 ± 0.37		18.34 ± 0.49		20.03 ± 0.55		21.19 ± 0.82
			MAE (A.U.)		30.3 ± 3.2		48.5 ± 3.0		70.5 ± 5.4		83.4 ± 4.8
			q		48		27		17		13

File size reduction (FSR), signal-to-noise ratio (SNR), maximum absolute error (MAE) and quality (*q*) for lossy compression. Literature values reported in [10] are shown for biceps brachii (BB). Mean values ± standard deviation were computed for 40 epochs per contraction level (% of MVC) of upper trapezius (UT) signals and for 8 epochs per contraction level (% of MVC) of medial gastrocnemius (MG) signals. Arrangements were longitudinal (L) and transversal (T).

Even though the experimental parameters—muscle, sampling frequency, inter-electrode distance, amplifier’s bandwidth and epoch size—are different from our work, results from [25] are used for comparison with ours, because they are the only values provided by the literature for compression of EMG signals recorded by two-dimensional electrode matrices. Literature results reported in [10] and [25] were obtained by linear prediction (ACELP), independent coding (IC), spectral prediction (SP), and space-and-time prediction (STP).

In lossy JPEG, reconstruction errors and compression ratios varied with the image quality. Lower qualities implied smaller files (greater FSRs) at the expense of lower SNRs. As a consequence, in order to obtain fair comparisons, lossy JPEG results were reported for the FSRs that were nearest to the three values provided by [10] and [25]—87.3%, 89.6% and 91.0%.

#### *Comparisons between lossy JPEG and algebraic-code excited linear prediction*

Literature results from [10] provide SNR results for an average FSR of 87.3%. Compression of propagating single-differential EMG signals from BB muscle by algebraic-code excited linear prediction (ACELP) provided mean SNR values of 10.38 dB (for 10%MVC), 11.81 dB (for 30%MVC), 12.25 dB (for 60% MVC), and 12.79 dB (for 70%MVC).

For the same FSR, lossy JPEG provided average SNR of 17.17 dB (for 20%MVC) and 18.63 dB (for 40%MVC) in longitudinally-arranged propagating single-differential signals from UT muscle. The transversal arrangement provided increases in SNR of 1.98 dB (for 20%MVC) and 1.82 dB (for 40%MVC) on average, in comparison to the longitudinal arrangement.

For the longitudinal arrangement of non-propagating monopolar signals from MG muscle, lossy-JPEG compression provided mean SNR values of 19.48 dB (for 20%MVC), 19.98 dB (for 40%MVC), 21.88 dB (for 60%MVC) and 23.33 dB (for 80%MVC). The transversal arrangement resulted in increases of 0.44 dB (for 20%MVC), 1.01 dB (for 40%MVC), 1.07 dB (for 60%MVC) and 0.91 dB (for 80%MVC) on mean SNR values.

**Figure 9:**
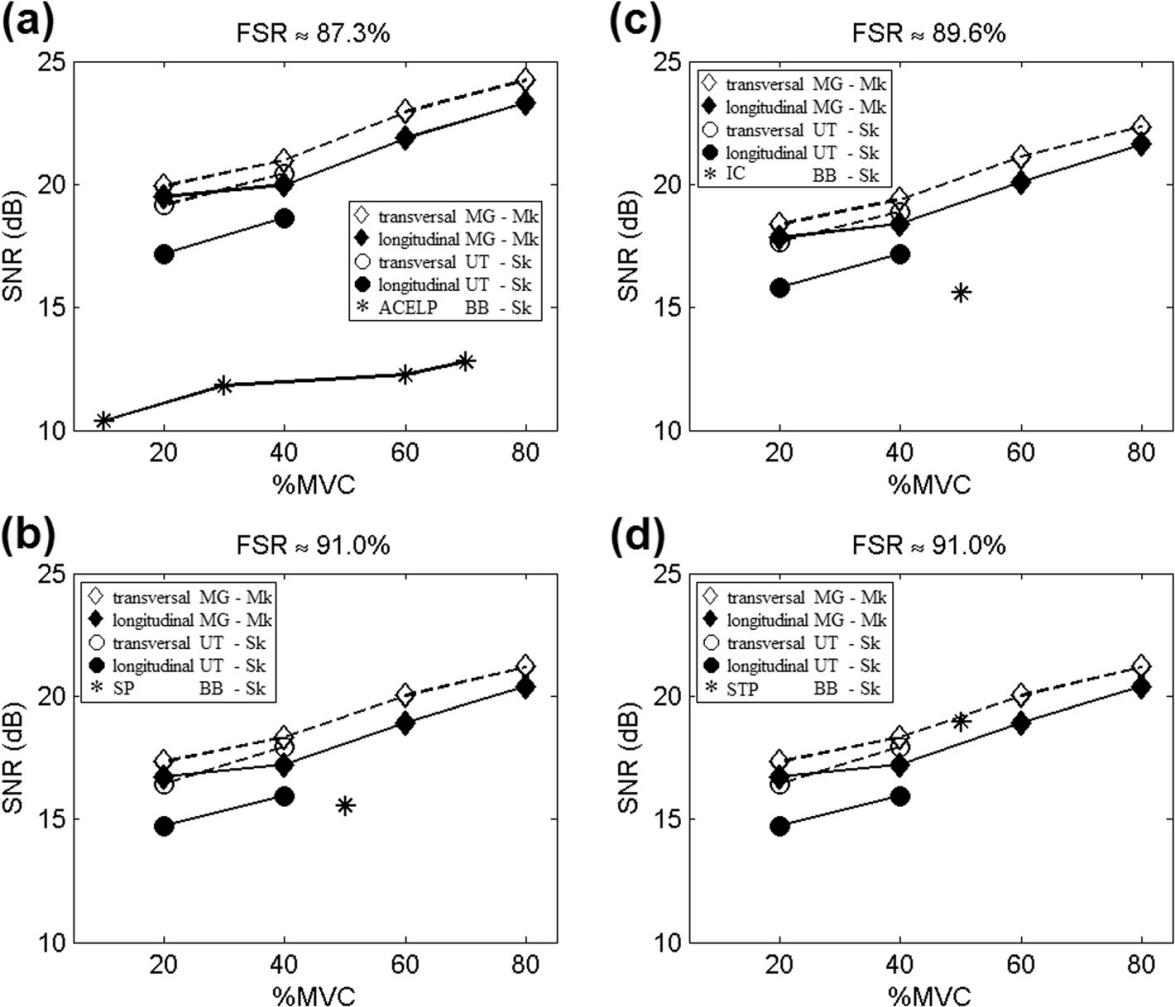
**Comparison with literature results.** Signal-to-noise ratio (SNR) in decibels (dB) as functions of contraction level (%MVC). Lossy-JPEG compression SNRs are shown for medial gastrocnemius signals in transversal (empty diamonds) and longitudinal (filled diamonds) arrangements, and for upper trapezius signals in transversal (empty circles) and longitudinal (filled circles) arrangements. Literature results (stars) are given for biceps brachii signals, using the following compression methods: **a)** algebraic-code excited linear prediction (ACELP) for an 87.3% FSR, **b)** independent coding (IC) for an 89.6% FSR, **c)** spectral prediction (SP) for a 91.0%FSR, and d) spatial and temporal codebook-excited linear prediction (STP) for a 91.0% FSR.

Figure 9a presents mean SNR values provided by the two compression methods (ACELP and lossy JPEG), for a FSR of 87.3%.

#### *Comparisons between lossy JPEG and independent coding*

Literature results from [25] provide SNR results for a FSR of 89.6%. Compression of propagating single-differential EMG signals from BB muscle by independent coding (IC) provided an average SNR of 15.58 dB (for 50%MVC).

For the same FSR, lossy JPEG provided an average SNR of 15.81 dB (for 20%MVC) and 17.17 dB (for 40%MVC) in longitudinally-arranged signals from UT muscle. The transversal arrangement increased the average SNR by 1.85 dB (for 20%MVC) and 1.70 dB (for 40%MVC), in comparison to the longitudinal arrangement.

For the longitudinal arrangement of monopolar signals from MG muscle, lossy-JPEG compression provided an average SNR of 17.84 dB (for 20%MVC), 18.39 dB (for 40%MVC), 20.09 dB (for 60%MVC), and 21.63 dB (for 80%MVC). The transversal arrangement of these signals resulted in increases of 0.53 dB (for 20%MVC), 1.00 dB (for 40%MVC), 1.05 dB (for 60%MVC), and 0.73 dB (for 80%MVC) on average.

Figure 9b presents the SNR for a FSR of 89.6%, using independent coding and lossy JPEG.

#### *Comparisons between lossy JPEG and spectral prediction*

Literature results from [25] also provide SNR results for a FSR of 91.0%. Compression of propagating single-differential EMG signals from BB muscle by spectral prediction (SP) provided a SNR of 15.55 dB (for 50%MVC).

For the same FSR, lossy JPEG provided mean SNR values of 14.72 dB (for 20%MVC) and 15.94 dB (for 40%MVC) in longitudinally-arranged signals from UT muscle. The transversal arrangement of these signals resulted in SNR increases of 1.72 dB (for 20%MVC) and 2.00 dB (for 40%MVC) on average, in comparison to the longitudinal arrangement.

For the longitudinal arrangement of monopolar signals from MG muscle, lossy-JPEG compression provided mean SNR values of 16.74 dB (for 20%MVC), 17.23 dB (for 40%MVC), 18.92 dB (for 60%MVC) and 20.39 dB (for 80%MVC). The transversal arrangement resulted in increases of 0.60 dB (for 20%MVC), 1.11 dB (for 40%MVC), 1.11 dB (for 60%MVC) and 0.80 dB (for 80%MVC) on average, for the lossy-JPEG compression of the same signals.

For a 91.0% FSR, the SNR results are presented in Figure 9c. Compression methods are spectral prediction and lossy JPEG.

#### *Comparisons between lossy JPEG and space-and-time prediction*

Literature results from [25] also provide SNR results for a different compression method for the same FSR of 91.0%. Compression of propagating single-differential signals from BB muscle by space-and-time prediction (STP) provided average SNR of 18.96 dB (for 50%MVC).

The results for lossy JPEG for the same FSR have already been presented in the subsection that showed comparisons with spectral prediction.

Figure 9d shows the average SNR for a 91.0% FSR. The results are presented for space-and-time prediction and lossy JPEG.

## Discussion

Compared to lossy compression, lossless methods provided lower compression ratios, as expected. However, they provide an important option whenever reconstruction errors are not admissible. Applications involving surface EMG decomposition into the constituent trains of motor unit action potentials require lossless compression, even at the expense of smaller FSR. On the other hand, applications concerning the display of EMG amplitude maps to identify regions of different activity levels can tolerate lower signal-to-noise ratios.

Lossy methods were applied only to the matrices of longitudinal and transversal arrangements of the original recorded signals, not to the signals obtained from differentiation. If differentiation had been used, decompression errors would have been introduced on the sample differences. Since the signals would be reconstructed by accumulating these sample differences, errors would increase as a function of time *t* or signal number *k*. For example, the last samples reconstructed from time differences would have an error whose variance would be over 1,000 times the variance for the first sample on the same 500 ms epoch.

### Effect of time-differentiation on lossless compression

Time differentiation not only improved the FSR of lossless JPEG compression—regardless of the signal arrangement—but also delivered the highest FSR for propagating single-differential signals recorded from UT muscle at 20%MVC and 40%MVC.

The effect of time-differentiation on non-propagating single-differential signals was not favorable for the lowest contraction force (20%MVC) of the MG muscle. However, it showed its usefulness for higher contraction forces (40%MVC to 80%MVC). One possible explanation follows.

Before the beginning of the acquisition, the EMG amplifier gain was adjusted so that EMG signals would not saturate for any contraction level, up to 80%MVC. As a consequence, at 20%MVC, monopolar signals from MG muscle were small in comparison to the AD converter full range, as shown in Figure 5a. As single-differential signals were computed, their amplitudes became even smaller as seen in Figure 5b, while maintaining the high predictability between neighboring samples that is used by lossless JPEG. When time-differences were computed on single-differential signals, the amplitude decreased even more. Furthermore, the predictability in time and between channels was negatively affected. Figure 5c shows that time-differentiated signals have higher frequency components than single-differential signals. This effect would account for the increase in file sizes, as compared to the single-differential signals, for 20%MVC. On the other hand, for higher contraction forces, this effect would be counterbalanced by the significant decrease on the amplitudes, shown in Figure 5e and 5f, resulting in better compression performance.

### Effect of transversal arrangement on lossless compression

For lossless JPEG compression, no significant improvement was attained by using the transversal arrangement in comparison to the longitudinal arrangement. For propagating signals in the longitudinal arrangement (UT muscle), *S*_2_(*t*) could be seen as a delayed version of *S*_1_(*t*). It could be expected that *S*_1_(*t*) would provide better estimates for its non-delayed version *S*_23_(*t*) —nearest neighbor signal in the transversal arrangement—than for its delayed version *S*_2_(*t*) —which is the nearest neighbor signal in the longitudinal arrangement. However, results show that ${\hat{S}}_{2}\left(t\right)$ is well determined by the weighted sum of *S*_2_(*t* - 1), *S*_1_(*t*) and *S*_1_(*t* - 1), which are the neighbor samples used by lossless JPEG in the longitudinal arrangement. This would be a plausible explanation for the indifference of lossless-JPEG compression to the longitudinal or transversal arrangements.

However, other methods take profit from the transversal arrangement. Used in combination with lossless ZIP compression of propagating single-differential signals, the transversal arrangement improved the FSR by 1.80% (for 20%MVC) and 2.00% (for 40%MVC) in comparison to the longitudinal arrangement, without introducing noise. It also provided additional FSRs that varied from 0.79% (for 20%MVC) up to 1.47% (for 80%MVC), for non-propagating single-differential signals. The transversal placement of EMG signals on images increased the spatial correlation between neighbor signals and consequently reduced the differences between them. This fact improved the compression performance for lossless ZIP compression of both propagating and non-propagating single-differential signals.

### Effect of transversal arrangement on lossy compression

Compared to the longitudinal arrangement, the transversal arrangement caused improvements in the SNR, using lossy JPEG compression of EMG signals at all contraction forces. The transversal arrangement of EMG signals resulted in increases of the SNR that varied from 0.44 dB to 2.00 dB, as compared to the longitudinal arrangement of the same signals.

Results suggest that, in order to achieve the highest compression ratios with lossy JPEG compression, data should be placed in an image where each row represents one of the propagating single-differential signals varying in time, with the signal-placement order established by sweeping the electrode matrix in the direction perpendicular to the muscle fibers. Similarly, non-propagating monopolar signals should be placed in an image whose placement order is provided by the direction perpendicular to the muscle longitudinal axis.

For propagating single-differential signals, the increase in SNR was more marked than for non-propagating monopolar signals. The transversal arrangement of signals takes advantage of the high spatial correlation between the rows of the electrode matrix. For propagating signals, the transversal arrangement induces the clustering of similar signals in the image, with no delay between them. Since lossy JPEG is computed by the DCT, this clustering allows the concentration of the largest DCT components in the lowest frequencies. The low-frequency DCT components are less affected by quantization, so smaller reconstruction errors are obtained. Consequently, the transversal arrangement improves the FSR in lossy JPEG compression of propagating signals, as compared to the longitudinal arrangement. For non-propagating signals, the transversal arrangement would not bring such an advantage. Since the fibers of the MG muscle are not parallel to the skin or to the longitudinal axis of the muscle, the surface signals show no propagation in the direction of the rows of the electrode matrix. So, the correlation between channels is different with respect to the case where the muscle fibers are parallel to the rows of the electrode array. As a consequence, the transversal arrangement is expected not to achieve the same results as with the propagating signals. Indeed, the transversal arrangement resulted in SNR increases in the range of 0.44 dB to 1.11 dB for the non-propagating MG signals, as compared to increases in the range of 1.72 dB to 2.00 dB for the propagating UT signals.

### Comparisons between lossy JPEG and literature results

Carotti and colleagues [10,25] reported compression results with acceptable reconstruction errors from the BB muscle (non-pinnate muscle with fibers parallel to the skin). Several compression techniques described in their work resulted in SNR values from 10.38 dB to 18.96 dB, for FSRs in the range of 87.3% to 91.0%, and contraction forces varying from 10%MVC to 70%MVC.

In our work, the transversal arrangement of EMG signals in images and their lossy-JPEG compression provided SNRs in the range of 16.44 dB to 24.24 dB, for FSR from 87.2% to 90.8%, for contraction forces varying from 20%MVC to 80%MVC.

Our results are therefore better than those provided by ACELP coding applied to individual EMG signals [10], to independent channels [25] and to spectral prediction [25]. Our values are also comparable to the results from spatial and temporal codebook-excited linear prediction [25], with the advantage of being obtained through a public-domain algorithm for image compression, whose computational time is smaller than for linear prediction methods.

### Future work

As the technique of “EMG Imaging” evolves towards larger electrode arrays, the issue of wireless transmission of many signals using a limited bandwidth becomes more and more relevant.

This work showed the influence of pre-processing procedures in the compression performance, such as the order (longitudinal or transversal) in which signals are placed into images and the usefulness of space and time-differentiation. It also focused on the differences of propagating and non-propagating EMG signals (note that time and space differentiation are equivalent for propagating signals but not for non-propagating signals). In this work, several parameters were kept constant, in order to allow comparisons between UT and MG muscles.

The increase of sampling frequency in time and space (reduction of inter-electrode distance) above the Nyquist rate should improve the compression performance, at the expense of adding redundant information and increasing the original file sizes. Even though the FSR could benefit from such changes, they would increase not only the non-compressed-file sizes but also the compressed-file sizes. A comprehensive study on the effects of varying sampling frequency and inter-electrode distance could generate a future work, with the aim of determining the optimal choice of both parameters. This study would allow increasing the inter-sample interval and the inter-electrode distance, without compromising the EMG characteristics of interest such as spectral content or time resolution.

Another parameter that affects JPEG performance is the image size—for similar contents, larger images usually result in better compression performance. Recording signals for longer intervals before applying data compression and transmission could be profitable. As a consequence, the interplay between sampling frequency and epoch duration could be the focus of future research.

Furthermore, EMG-signal morphology varies with different muscles, subjects and pathologies. A signal compression technique must be suitable for all cases and conditions. Two very different types of healthy muscles have been considered in this work. Further work is warranted to verify the effectiveness of the technique in extreme cases.

## Conclusions

This work provided SNR and FSR values for lossy compression of propagating and non-propagating EMG signals. For a FSR of 90.8%, SNRs of 17.94 dB (for propagating single-differential signals at 40%MVC) and 21.19 dB (for non-propagating monopolar signals at 80%MVC) were attained. For a smaller FSR of 87.2%, SNR reached even higher values—20.45 dB (for propagating signals at 40%MVC) and 24.24 dB (for non-propagating signals at 80%MVC).

Whenever very high SNR values are the goal, one should consider the possibility of using lossless compression, at the expense of lower FSRs. Reference values of FSR for lossless JPEG compression of single-differential EMG signals in the transversal arrangement are provided for various contraction force levels, in Table 1. For UT single-differential signals, the reference FSR values are 40.88% (for 20%MVC) and 38.15% (for 40%MVC). For MG single-differential signals before time-differentiation FSR is 59.30% (for 20%MVC), and after time-differentiation FSRs are 53.41% (for 40%MVC), 49.90% (for 60%MVC) and 48.22% (for 80%MVC).

The transversal placement of multi-channel EMG signals on images is a simple procedure that improves the compression performance of lossless-ZIP and lossy-JPEG methods, and causes no harm to lossless-JPEG compression. Furthermore, the time-differentiation of single-differential signals before lossless-JPEG compression should be considered.

For online compression problems, the use of fast image-compression algorithms may be of significant help, as long as the methodological suggestions are followed.

## Competing interests

There are no competing interests.

## Authors’ contributions

CI prepared the manuscript and implemented the methods computationally. CI proposed the use of transversal and longitudinal arrangements, time-differentiation and JPEG compression. SSF proposed the use of ZIP compression and MAE. RM proposed the use of non-pinnate and pinnate muscles, and provided the EMG signals. SSF and RM revised and gave the final approval of the manuscript. All authors read and approved the final manuscript.
